# Assessment of inflammatory resilience in healthy subjects using dietary lipid and glucose challenges

**DOI:** 10.1186/1755-8794-6-44

**Published:** 2013-10-27

**Authors:** Suzan Wopereis, Danielle Wolvers, Marjan van Erk, Michiel Gribnau, Bas Kremer, Ferdi A van Dorsten, Esther Boelsma, Ursula Garczarek, Nicole Cnubben, Leon Frenken, Paul van der Logt, Henk FJ Hendriks, Ruud Albers, John van Duynhoven, Ben van Ommen, Doris M Jacobs

**Affiliations:** 1 Unilever R&D, Olivier van Noortlaan 120, Vlaardingen 3130 AC, The Netherlands; 2 TNO, Utrechtseweg 48, Zeist 3704 HE, The Netherlands; 3 Netherlands Metabolomics Centre, Einsteinweg 55, 2333 CL, Leiden, The Netherlands; 4 NutriLeads Innovations, Pancrasgorsedijk 7, Rockanje 3235 KT, The Netherlands; 5 Wageningen University, Dreijenlaan 3, Wageningen 6703 HA, The Netherlands

**Keywords:** OGTT, OLTT, Challenge test, Inflammation, Resilience, Oxylipins, Lipid, Glucose

## Abstract

**Background:**

Resilience or the ability of our body to cope with daily-life challenges has been proposed as a new definition of health, with restoration of homeostasis as target resultant of various physiological stress responses. Challenge models may thus be a sensitive measure to study the body’s health. The objective of this study was to select a dietary challenge model for the assessment of inflammatory resilience. Meals are a challenge to metabolic homeostasis and are suggested to affect inflammatory pathways, yet data in literature are limited and inconsistent.

**Method:**

The kinetic responses of three different dietary challenges and a water control challenge were assessed on various metabolic and inflammatory markers in 14 healthy males and females using a full cross-over study design. The dietary challenges included glucose (75 g glucose in 300 ml water), lipids (200 ml whipping cream) and a mix of glucose and lipids (same amounts as above), respectively. Blood samples were collected at baseline and at 0.5, 1, 2, 4, 6, 8 and 10 h after consumption of the treatment products. Inflammation (IFNγ, IL-1β, IL-6, IL-8, IL-10, IL-12p70, TNF-α CRP, ICAM-1, VCAM-1, SAA, E-selectin, P-selectin, thrombomodulin, leukocytes, neutrophils, lymphocytes) and clinical (e.g. glucose, insulin, triglycerides) markers as well as gene expression in blood cells and plasma oxylipin profiles were measured.

**Results:**

All three dietary challenges induced changes related to metabolic control such as increases in glucose and insulin after the glucose challenge and increases in triglycerides after the lipid challenge. In addition, differences between the challenges were observed for precursor oxylipins and some downstream metabolites including DiHETrE’s and HODE’s. However, none of the dietary challenges induced an acute inflammatory response, except for a modest increase in circulating leukocyte numbers after the glucose and mix challenges. Furthermore, subtle, yet statistically significant increases in vascular inflammatory markers (sICAM-1 and sVCAM-1) were found after the mix challenge, when compared to the water control challenge.

**Conclusions:**

This study shows that dietary glucose and lipid challenges did not induce a strong acute inflammatory response in healthy subjects, as quantified by an accurate and broad panel of parameters.

## Background

The World Health Organization (WHO) defines health as “a state of complete physical, mental and social well-being and not merely the absence of disease and infirmity” [1]. Recently, it has been suggested to change the emphasis of this definition “towards the ability to adapt and self-manage in the face of social, physical and emotional challenges” [2].

This concept of resilience links health to the body’s capacity to deal with daily stressors that challenge our homeostasis, with restoration of homeostasis as target resultant of various physiological systems [3]. In order to comply with this newly proposed definition, experimental models need to be developed that describe the disturbance and restoration of homeostasis (challenge models) and that can thus be employed as measures of health and wellbeing.

This development of challenge models is attractive for nutritional research increasingly focusing on the maintenance and improvement of health. Nutrition impacts health every day by inducing subtle and pleiotropic effects that are not readily detectable using static homeostatic measures [4]. The kinetic response to a homeostatic perturbation is hypothesized to be a more sensitive measure for detecting effects of nutritional interventions. Moreover, comprehensive multi-parametric (“omics”) analysis measured under conditions of physiological stress may identify key parameters that are more adequate to describe healthy and compromised conditions when compared to current biomarkers, which are typically assessed during steady state and regarded as markers of disease.

The concept of resilience is relevant for metabolic and inflammatory processes, because these processes are continuously challenged and need optimal adaptive flexibility to maintain optimal health. Metabolic and inflammatory processes are highly interrelated. Excessive amounts of glucose and lipids induce metabolic stress that is sensed by metabolic cells such as adipocytes and immune cells such as macrophages, leading to the induction of oxidative and inflammatory responses [5,6]. The close functional and molecular integration of the immune and metabolic systems is emerging as a crucial homeostatic mechanism, the dysfunction of which underlies many chronic metabolic diseases, including type 2 diabetes and atherosclerosis.

The aim of the present study was to investigate the ability of healthy subjects to restore inflammatory homeostasis after consumption of high energy dietary loads. Models of postprandial inflammation were considered as a good starting point to study the inflammatory resilience, considering the current evidence of inflammatory responses after a dietary challenge in both healthy and metabolically compromised subjects [7-14]. However, data are conflicting. Derosa *et al*. have shown that a standardized oral fat load as well as oral glucose tolerance test (OGTT) induced significant changes in inflammatory response markers and markers of endothelial dysfunction (hsCRP, IL-6, TNF-α, sICAM-1, sVCAM-1 and sE-selectin) in adult healthy subjects [15,16]. However, other studies have failed to demonstrate effects on inflammatory markers (IL-6, TNF-α and hsCRP) upon a high-fat dietary load in healthy men [17,18].

Despite the complex regulation of metabolic and inflammatory pathways, the postprandial inflammatory response to different nutrients have mostly been assessed using only a limited set of inflammatory markers such as TNF-α, IL-6 and hsCRP. The response to single bolus ingestions of glucose and fat have not been compared in a single study using a broad range of inflammatory mediators (cytokines, adhesion molecules, acute phase proteins, haematology, oxylipins) and inflammation related gene expressions in blood cells.

In this explorative, complete cross-over study, we compared side-by-side the postprandial inflammatory response upon three dietary challenges (glucose, lipids, mix of glucose and lipids) in healthy subjects using a multi-parametric analytical approach. We hypothesized that glucose and lipids will affect different inflammatory pathways eventually leading to differential gene expression and levels in inflammatory mediators and that a mix of glucose and lipids will elicit the strongest inflammatory response. A water challenge was included to account for any diurnal, prolonged fasting or other experiment-related effects.

## Methods

### Participants

This study is an exploratory study designed to investigate the response of many parameters upon different dietary challenges. As none of these parameters was the clear-cut, unique endpoint, the sample size was based on a limit for the desired information increase per additional subject. This statistical consideration has been outlined by Julious [19] and resulted in a sample size of 14 subjects per group, permitting for two possible drop-outs.

Thus, six males and eight females were recruited from the pool of volunteers of TNO, Zeist. They were apparently healthy adults with the following mean ±SEM characteristics: age, 54 ± 6 years; BMI, 22 ± 1 kg/m^2^; and waist circumference, 80 ± 8 cm. The demographic characteristics of the study participants at inclusion and the average baseline (t = 0 h) levels of the clinical parameters are listed in Table 1 and Additional file 1: Table S1, respectively. Exclusion criteria were any history of medical or surgical events, any chronic disease related to inflammation or allergy, chronic use of medication that affect inflammatory processes (e.g. Aspirin, antibiotics), regular use of lipid lowering medications and/or cholesterol lowering products (e.g. *Becel Pro-activ*), lactose intolerance or other food allergies, alcohol consumption (> 28 units/week for males and > 21 units/week for females), smoking, extreme physical exercise, reported slimming or medically prescribed diet, recent blood donation (< 1 month prior to the start of the study), and for women pregnancy or lactating. The study protocol was approved by the independent medical ethics committee of TNO METOPP (Tilburg, The Netherlands) and all subjects gave written informed consent.

**Table 1:** Clinical parameters at baseline (t = 0)

	**OGTT**		**OLTT**		**OL + GTT**		**Control**	
	**(n** = **14)**		**(n** = **14)**		**(n** = **14)**		**(n** = **14)**	
**Compound**	**Mean**	**std**	**Mean**	**std**	**Mean**	**std**	**Mean**	**std**
Glucose [mmol/L]	5.51	0.42	5.46	0.53	5.44	0.61	5.46	0.56
Insuline [mU/L]	4.8	2.3	4.2	1.5	3.8	2.1	3.8	1.5
Cholesterol (total) [mmol/L]	5.44	0.95	5.60	0.98	5.38	1.00	5.38	0.98
HDL cholesterol [mmol/L]	1.63	0.26	1.64	0.31	1.61	0.29	1.65	0.29
LDL cholesterol [mmol/L]	3.3	0.9	3.4	0.9	3.3	0.9	3.2	0.9
Ratio cholesterol/HDL	3.4	0.8	3.5	0.8	3.4	0.8	3.3	0.8
Triacylglycerides [mmol/L]	1.22	0.43	1.16	0.42	1.11	0.49	1.13	0.43
Gamma-GT [U/L]	18.9	9.5	18.5	8.2	17.7	8.6	18.2	8.3
ALAT [U/L]	11	5	11	4	11	6	12	6
ASAT [U/L]	23	9	23	6	27	11	25	10
ALP [U/L]	60	19	59	18	57	17	60	18
Creatinine [μmol/L]	72	10	73	11	73	11	74	11
Urea [mmol/L]	5.4	1.2	5.4	1.0	5.5	1.0	5.8	1.5
RBC [tera/L]	4.59	0.34	4.60	0.31	4.53	0.36	4.60	0.38
Platelets [giga/L]	245	41	248	47	245	44	254	53
Reticulocytes [%]	0.79	0.21	0.77	0.23	0.80	0.27	0.76	0.17
Leucocytes [giga/L]	5.2	1.2	5.0	0.7	4.9	0.9	5.2	0.9
WBC: lymphocytes [%]	35.3	3.7	36.0	6.0	36.3	4.3	36.5	4.2
WBC: neutrophils [%]	54.8	5.8	54.2	7.1	53.6	4.4	53.7	4.7
WBC: monocytes [%]	5.8	1.7	5.9	1.4	6.3	1.5	5.9	1.3
WBC: eosinophils [%]	3.2	1.5	3.2	1.2	3.1	1.1	3.1	1.2
WBC: basophils [%]	0.8	0.3	0.6	0.2	0.7	0.3	0.8	0.2
Total bilirubin [μmol/L]	8.5	3.2	9.7	3.6	8.5	4.6	8.3	3.7
Uric Acid [μmol/L]	262	49	259	52	262	43	263	46
CRP [ng/mL]	736.3	503	634.0	399	918.6	1048	993.1	932
E-selectin [ng/mL]	12.4	3	13.1	3	12.6	4	13.1	4
IFN-gamma [pg/mL]	2.2	2	2.4	3	2.3	3	2.5	3
IL-10 [pg/mL]	198.7	456	187.8	471	188.2	513	211.1	500
IL-12p70 [pg/mL]	336.2	908	269.6	698	335.1	1042	354.1	957
IL-1β [pg/mL]	0.6	1	0.5	1	0.5	1	0.6	1
IL-6 [pg/mL]	1.1	1	1.2	1	1.1	1	1.3	1
IL-8 [pg/mL]	2.2	1	2.6	1	2.4	1	2.5	1
P-selectin [ng/mL]	44.4	16	48.1	14	48.7	19	50.9	20
SAA [ng/mLl]	2117.6	1646	1682.0	795	1924.4	1268	1959.8	1541
TNF-α [pg/mL]	3.6	1	3.8	1	3.9	1	4.2	2
Thrombomodulin [ng/mL]	4.7	1	4.7	1	4.7	1	4.8	1
sICAM-1 [ng/mL]	275.1	34	259.3	38	250.7	43	257.0	41
sICAM-3 [ng/mL]	2.7	1	2.8	2	2.8	1	2.9	1
sVCAM-1 [ng/mL]	442.4	87	440.8	79	419.7	72	448.7	113

### Study design

The subjects were tested on four days separated by one-week intervals. The total duration of the study was 22 days. During the whole study period, subjects were asked to maintain their habitual diet and normal physical activities. On the evening prior to each test day, subjects consumed a standardized meal of ~1000 kcal between 18:00–19:00 h and a snack between 21:00–22:00 h. After 22:00 h, they were not allowed to eat. They were only allowed to to drink water. The subjects were randomly allocated to a study treatment, with randomization restricted to waist circumference and age. The challenges were applied according to a Latin square design [20]. On each test day, the subjects arrived at the research facility in the morning after an overnight fast of 10 hours. A cannula was placed into a vein in the forearm for collection of blood samples. The cannula was kept patent by a saline solution infusion over the entire challenge period of 10 hours. After collection of baseline blood samples (t = 0), subjects consumed one of the challenge test products within 10 min. Blood samples were collected at regular time points (0, 0.5, 1, 2, 4, 6, 8, 10 h) up to 10 h. Two time points (0.5 and 1 h) were sparse sampled (half of the subjects sampled at 0.5 h and the other half at 1 h) to increase the resolution of the population means. No other foods or beverages were allowed during the day, except water.

Blood samples were collected in tubes containing EDTA as anti-coagulant. For the gene expression analysis PAXgene tubes were used. Whole blood was centrifuged for 10 min at 4°C at 13,000 rpm within 15 min of collection. Serum and plasma were immediately separated, aliquoted and stored at -80°C. For the oxylipins an inhibitor cocktail consisting of butylated hydroxytoluene (BHT), indomethacin, paraoxon, 12-[(tricyclo[3.3.1.13,7]dec-1-ylamino)carbonyl]amino]-dodecanoic acid and phenylmethylsulfonyl fluoride [21] was directly added to the plasma samples before storage.

### Dietary challenges

Four challenges were applied. The first challenge test (OGTT) was 75 g glucose (300 kcal) dissolved in 300 mL water and thus was identical to the intake used in a standardized oral glucose tolerance test. The second challenge test (OLTT) was 200 mL whipping cream (686 kcal; composition per 100 mL: 2.3 g proteins, 3.1 g carbohydrates, 35.6 g fat) filled up with water to 300 mL. The third challenge test (OG + LTT) was 200 mL whipping cream and 75 g glucose filled up with water to 300 mL. The total energy content of this challenge was 986 kcal. The dietary challenges were not isocaloric (i) to comply with the standardized condition of the OGTT challenge test and (ii) to be in line with other studies typically using >700 kcal for high-fat dietary challenges [15,17,22,23]. Furthermore, it was hypothesized that the dietary composition is a more crucial factor determining the inflammatory response than the caloric intake. The fourth challenge was a control challenge consisting of the intake of 300 mL water.

### Clinical chemistry and inflammatory marker measurements

Serum and EDTA-blood were collected for clinical chemistry tests and the measurement of a range of inflammatory markers. Serum clinical chemistry tests included the measurements of γ-glutamyltransferase, alanine aminotransferase, aspartate aminotransferase, alkaline phosphatase, glucose, insulin, total bilirubin, creatinine, urea, uric acid, total cholesterol, HDL cholesterol, LDL cholesterol, triglycerides, CRP, and haematology and were performed using Olympus analytical equipment and reagents (Olympus AU400 clinical chemistry analyzer; Olympus-Diagnostica Europe, Hamburg, Germany). The markers glucose, insulin, total cholesterol, HDL cholesterol, LDL cholesterol, triglycerides, CRP, total leucocytes and white blood cell differentiation parameters were used in the analysis of challenge test effects. The other clinical chemistry markers were used for screening of the health status of the study participants. Plasma samples were used for multi-array analyses of 7 inflammatory proteins: IFNγ, IL-1β, IL-6, IL-8, Il-10, Il12p70 and TNF-α (Mesoscale Discovery, Multiplex panel Human Proinflammatory 7-plex, Rockville, U.S.A.) and of 8 vascular proteins: CRP, ICAM-1, VCAM-1 and SAA (Mesoscale Discovery, Multiplex panel Human Vascular Injury II) and sICAM-3, E-Selectin, P-Selectin, and Thrombomodulin (Mesoscale Discovery, Multiplex panel Human Vascular Injury I). Most soluble biomarkers displayed plasma levels above the lower detection limit of the assay, except for IL-1β, which was below the lower detection limit of the assay in most subjects.

### Plasma extraction of eicosanoids

Samples from 12 subjects were included in the oxylipin analysis. The two subjects from the reserve list were excluded from this analysis. Plasma samples (250 μL) collected at six time points (0, 2, 4, 6, 8, 10 h) for oxylipin analysis were treated with methanol (1:5) and incubated for 30 min on ice. Samples were subsequently centrifuged (5 min at 3000 × *g* and 4°C) and the supernatant was transferred to a glass tube. Just before loading on activated hydrophilic-lypophilic balance (HLB) columns, 4.75 mL of Milli-Q purified water (MQ) containing 0.1% v/v of FA were added to the methanol extract, diluting the extract to 20% methanol. After loading, the columns were washed with 2 mL of 20% methanol in MQ water containing 0.1% of FA, and the columns were allowed to dry for 15 min. The solid phase extraction columns were eluted with 2 mL methanol and the samples were captured in tubes already containing 20 μL of 10% glycerol and 500 μM BHT in ethanol. The tubes were placed in a water bath at 40°C. The methanol was evaporated under a gentle stream of nitrogen, reconstituted in 100 μL ethanol containing another internal standard 1-cyclohexyl-3-doceanoic acid urea (CUDA) and immediately used for LC-MS/MS analysis.

### LC-MS/MS analysis of eicosanoids

The analysis was performed on a UPLC coupled to a Xevo TQ-S mass spectrometer (Waters). Five μL extract were injected on an Acquity C18 BEH UPLC column (2.1 × 100 mm, 1.7 μm) and separated using gradient elution with a stable flow of 600 μL/min. The gradient started with 95% A (MQ water with 0.1% FA) and 5% B (acetonitrile (ACN) with 0.1% FA) followed by a linear increase to 70% A and 30% B which was achieved at 5.00 min. This was followed by a linear increase towards 50% A – 50% B which was achieved at 11.25 min and maintained until 13.25 min. The system was subsequently switched to 100% B, which was achieved at 15.75 min and maintained until 16.75 min, after which the column was equilibrated at 95% A for approximately 3 min. The column was maintained at 50°C during analysis, and the samples were kept at 10°C. The MS was operating in selective reaction mode using electrospray ionization in negative ion mode, with a capillary voltage of 3.3 kV, a source temperature of 150°C and a desolvation temperature of 600°C. Cone voltage and collision energy were optimized for each compound individually, and parent and product *m/z* values are listed in Additional file 1: Table S2. Peak identification and quantification were performed using MassLynx software version 4.1. Calibration curves were run in duplicate from which one regression equation was generated. The calibration ranges differed, depending on the naturally occurring concentrations of the individual compounds in plasma, e.g. 234–15000 μg/L for DHA and 0.12-7.5 μg/L for 15 (S)-HETE. In order to limit the data processing, only compounds relevant for this study were selected. For this, a limited number of samples were pooled per time point and treatment and analyzed as initial batch. Only compounds detected in this initial batch were selected for further processing of the other batches (shown in bold in Additional file 1: Table S2).

### Quality control of LC-MS/MS analysis of eicosanoids

The samples were analyzed in 8 batches. Each batch contained 42 samples and 6 quality control samples prepared from a pooled plasma sample. The quality control samples were used to determine the precision and accuracy for all compounds reported in this study. The precision and accuracy were comparable to results published by Balvers *et al*. [21].

### Gene expression

Samples from 12 subjects were included in the gene expression analysis. The two subjects from the reserve list were excluded from this analysis. Blood samples were collected in PAXgene tubes (Qiagen/BD) at 0, 2 and 6 h for each challenge. RNA was isolated using PAXgene blood RNA kit (Qiagen) according to manufacturer’s instructions. Next, 1 μg of RNA was converted into cDNA using High Capacity RNA-to-cDNA kit (Applied Biosystems) and diluted to 10 ng/μL. Realtime PCR was performed by ServiceXS B.V. (Leiden, the Netherlands) on Fluidigm’sBioMark 96.96 Dynamic Arrays for Gene Expression, measuring expression of 96 genes in 96 samples. The genes were selected based on existing knowledge of their role in inflammation and based on expression above background in blood cells based on previous studies [24,25]. The selected genes included in the analysis are listed in Additional file 1: Table S3.

The cDNA samples were subjected to 14 cycles of specific target amplification (STA), using a cocktail of all combined Gene Expression primer sets and the TaqmanPreAmp Master Mix (Applied Biosystems). Water was included as a no-template control (NTC). The NTCs were also included in the STA reaction, to serve as a true negative control for the entire procedure. After 5 fold dilution, the STA samples were used on a BioMark 96.96 Dynamic Array for Gene Expression, for determination of Ct (cycle threshold) values. Pair-wise combinations of all samples were made with each of the assays on each array. The default EvaGreen PCR protocol was used on the BioMark instrument with an annealing temperature of 60°C and a total of 35 cycles of PCR. The PCR was followed by Melting Curve Analysis. Melting was monitored between 60 and 95°C.

The BioMark Real-Time PCR Analysis software version 3.0.2 was used for Ct determination from the 9216 reaction chambers on each array and for the analysis of melting curve data (default settings). The baseline correction chosen was 'Linear’ in combination with the 'User (Detectors)’ Ct threshold method, using the option 'Initialize with Auto’.

For each gene, a dilution series was measured using a pooled sample. This dilution series was used to assess the relative concentrations of each gene which were then corrected for the relative concentration of housekeeping gene ubiquitin C (UBC). Eight genes did not pass quality control: CCL20, CXCL2, CYP4A11, MRC1, PTGIS, EMP1, AKR1C3, and NOS2. Furthermore, two alternative housekeeping genes (GAPDH and YWHAZ) were included on the array but not considered for further analysis because of the better performance of the housekeeping gene UBC. Thus, a total of 85 genes were quantified and used to assess the effect of the different challenges. They were analyzed in Ingenuity (Ingenuity Systems Inc, Redwood City, CA, USA) and enriched in biological functions related to inflammatory response (#72 out of 85 genes), cellular movement (#65 out of 85 genes) and immune cell trafficking (#63 out of 85 genes). Based on top networks, and canonical pathways different gene-sets were created related to specialized biological functions and pathways: lipid metabolism related to inflammatory response (n = 14); inflammatory response related to infectious disease (n = 11); lipid metabolism related to molecular transport (n = 11); organ development and lymphoid tissue (n = 11); antigen presentation and cellular movement (n = 10); IL-10 signaling (n = 17); atherosclerosis signaling (n = 18); peroxisome proliferator-activated receptor (PPAR) signaling (n = 15) and IL-6 signaling (n = 15). The genes belonging to these different gene-sets are summarized in Additional file 1: Table S4.

#### Data analysis

The kinetic response of the four different dietary challenges on various metabolic and inflammatory markers was assessed by determining delta values relative to baseline concentrations and by several area under the curve (AUC) measures calculated by the trapezoidal rule. First, the AUC and incremental AUC (iAUC) values corrected for the baseline measurement (t = 0) were calculated. Secondly, the iAUC under (AUC_min_) and over (AUC_plus_) the baseline measurement (t = 0) as well as their sum (AUC_total_) were calculated. A mixed model analysis was used to test for differences in AUC between the dietary challenges and the water control challenge (Additional file 1: Figure S1) with challenge, visit and cohort as fixed factors, baseline and baseline*challenge as covariates and the subjects as random factor, where the * refers to an interaction effect. Next, the challenge response curves of the different markers were compared by a repeated-measures analysis with challenge, visit, cohort, time, challenge*time, visit*time and challenge*time as fixed factors, baseline, baseline*challenge and baseline*time as covariates and the subjects as random factor, where the * refers to an interaction effect. For both analyses, the challenges were compared to the water control challenge using a 2-sided test adjusted by a Dunnett multi-comparison correction. The null hypothesis (no difference) was rejected when the p-value was below 0.05. If necessary, AUC and response curve data were log-transformed. For this, the Anderson-Darling test was used to test for normality; a log transformation was applied when the data were not normal. Statistical outliers, defined as a value that differed more than 3 times the standard deviation from the median value, were excluded for analysis. When visualizing average curves of treatments the outliers were replaced by the median response value of the corresponding individual. Data visualization of the individual and average response curves of each marker was performed using Tibco Spotfire 2.2 0 (Tibco Spotfire Inc., Somerville, MA, USA). All statistical analyses were performed using SAS 9.2 (SAS Institute Inc. Cary, NC, USA).

## Results

### Baseline characteristics of study population

#### *Clinical chemistry*

All subjects met our inclusion criteria. The demographic data and baseline (t = 0) clinical chemistry characteristics of the 14 subjects who participated in the study are given in Table 1 and Additional file 1: Table S1, respectively. No differences between baseline values were found between the different challenges. One subject had high fasting glucose levels (ranging between 5.9 and 6.5 mmol/L; normal values < 6.1 mmol/L) and two other subjects had high levels of total cholesterol (ranging from 6.2-7.2 and 6.9-7.4 mmol/L, respectively. Values > 6.2 mmol/L were considered as high risk).

#### *Inflammatory markers*

To assess the inflammatory response, a series of plasma markers i.e. CRP, SAA, sVCAM-1, sICAM-1, sICAM-3, IL-1β, IL-6, IL-8. IL - 10, IL-12p70, IFNγ, TNF-α, E-Selectin, P-Selectin, and thrombomodulin were measured. In addition, total leukocyte counts in plasma as well as lymphocytes, neutrophils, monocytes, eosinophils and basophils in white blood cells (WBC) were measured. The average baseline values and standard deviations for these markers are summarized in Table 1. The average baseline levels of cytokines, vascular adhesion molecules, SAA and CRP were not significantly different between the challenges, except for the baseline levels of IFNγ and TNF- α which were significantly lower prior to the OL + GTT and OGTT challenges, respectively, when compared to the control challenge.

All subjects had normal values of the inflammatory marker CRP ( i.e. < 10 mg/L) and the white blood cell counts (i.e. < 11 × 10E^9^/L). Therefore, we concluded that these subjects had no clinical inflammation. IL-10 and IL-12 showed the largest individual variation. Four out of 14 subjects had i.e. 1–2 orders of magnitude higher plasma concentrations of these cytokines. These high levels were consistently observed for the same subjects throughout the study. These 4 subjects also consistently displayed the highest levels of IFNγ, IL-1β, and TNF-α. No correlations were observed with other subject characteristics in these subjects.

#### *Oxylipins and gene expression*

No baseline differences were observed for oxylipins and most genes measured. The expression of the genes LCN2 (p = 0.050) and MAPK1 (p = 0.040) were marginally higher at OLTT baseline and the expression of PPARG was significantly (p = 0.0098) lower at OGTT baseline (1.7 fold difference from the control value).

### Effect of dietary challenges

#### *Clinical chemistry*

**Figure 1:**
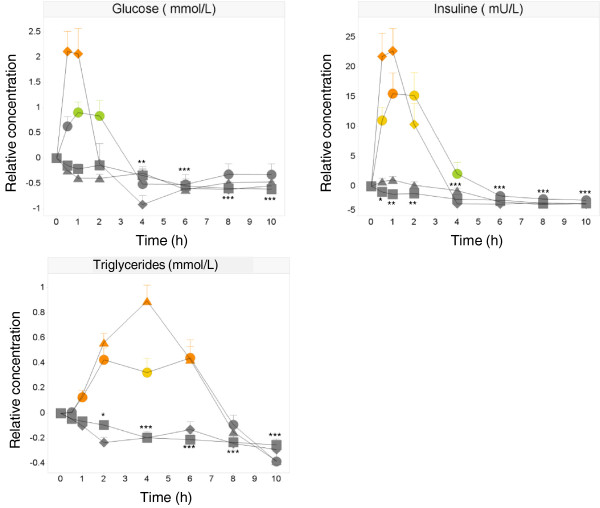
**Effect on glucose, insulin and triglycerides.** Mean ± SEM postprandial relative glucose, insulin and triglycerides in healthy subjects (N = 14) after ingestion of water (□), OGTT (◊), OLTT (△), OG + LTT (○). ANOVA: OGTT, OLTT, OL + GTT versus water: green p < 0.05, yellow p < 0.01, orange p < 0.001; water versus baseline; * p < 0.05, ** p < 0.01, *** p < 0.001.

The effect of different dietary challenges on a few clinical parameters (glucose, insulin, total cholesterol, HDL cholesterol, LDL cholesterol, and triglycerides) was investigated to check whether the challenges were successful. As expected the control challenge resulted in significantly decreased concentrations of most clinical chemistry parameters, when compared to baseline. Especially glucose (from 5.46 to 4.84 mmol/L), insulin (from 3.8 to 0.9 mU/L), and triglycerides (from 1.13 to 0.87 mmol/L) showed a strong linear decrease in plasma concentrations with lowest concentrations at 10 h due to up to 20 h of fasting (Figure 1).

The metabolic markers responded to the dietary challenges as expected: the glucose intake (OGTT) induced significantly different responses of plasma glucose and insulin when compared to the control challenge. Glucose concentrations increased at time points 0.5 h (8.26 mmol/L for OGTT vs 5.08 mmol/L for control) and 1 h (7.96 mmol/L for OGTT vs 5.45 mmol/L for control) and reached baseline levels at 4 h, whereas insulin showed increased concentrations at 0.5 h (34.6 mU/L for OGTT vs 2.3 mU/L for control), 1 h (27.7 mU/L for OGTT vs 2.6 mU/L for control) and 2 h (15.2 mU/L for OGTT vs 2.6 mU/L for control) (Figure 1).

Due to the lipid load (OLTT) the plasma insulin and triglyceride response was significantly different from the control curve (Figure 1). Insulin showed significantly higher concentrations at 0.5 h up to 4 h compared to control (9.4; 6.2; 4.3; and 3.4 mU/L for OLTT vs 2.3; 2.6; 2.6; and 1.6 mU/L for control). Triglycerides showed significant higher concentrations 2 h up to 6 h compared to control (1.73; 2.06; and 1.59 mmol/L for OLTT vs 1.04; 1.03 and 0.93 mmol/L for control) and baseline.

The combined lipid and glucose load (OG + LTT) induced significantly different responses of plasma insulin, glucose, and triglycerides when compared to the control challenge (Figure 1). Insulin showed significant higher concentrations at 1–4 h and 8 h (25.8; 19.0; 7.6 and 1.6 mU/L for OG + LTT vs 2.6; 2.6; 1.5 and 1.2 mU/L for control), and glucose had significant higher concentrations at 1–2 h and 8–10 h compared to control (7.09; 6.28; 5.12 and 5.12 mmol/L for OG + LTT vs 5.45; 5.32; 4.94 and 4.84 mmol/L for control). Triglycerides showed significantly higher concentrations at 2–6 h and significantly lower concentrations at 10 h (1.54; 1.56; 1.55 and 0.73 mmol/L for OG + LTT vs 1.03; 0.93; 0.92 and 0.87 mmol/L for control).

#### *Inflammatory markers*

**Figure 2:**
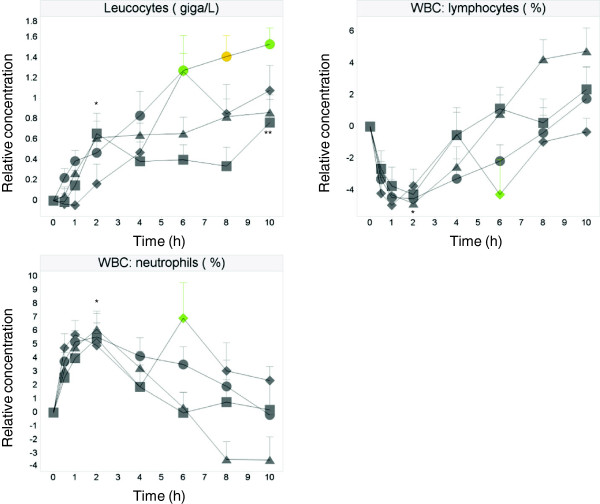
**Effect on leucocytes, lymphocytes and neutrophils.** Mean ± SEM postprandial relative leucocytes, lymphocytes and neutrophils in healthy subjects (N = 14) after ingestion of water (□), OGTT (◊), OLTT (△), OG + LTT (○). ANOVA: OGTT, OLTT, OL + GTT versus water: green p < 0.05, yellow p < 0.01, orange p < 0.001; water versus baseline; * p < 0.05, ** p < 0.01, *** p < 0.001.

Changes in leukocytes were observed upon all dietary challenges and the water control challenge. Total leukocytes displayed an increase in number over time, but stayed within normal clinical reference ranges (i.e. <11 × 10^9^/L). Statistically significant increases compared to baseline occurred at 2 h and 10 h after the control challenge, at 2, 4, 6, 8 and 10 h after both OLTT and OG + LTT, and at 6, 8 and 10 h after OGTT (Figure 2). Between-challenge comparisons showed that the increases in leukocyte numbers were significantly different from the control challenge at 6 h after the OGTT challenge (6,5 × 10^9^/L for OGTT vs 5.6 × 10^9^/L for control, p < 0.01) and at 6 h (6.1 × 10^9^/L for OG + LTT vs 5.6 × 10^9^/L for control, p < 0.05), 8 h (6.2 × 10^9^/L for OG + LTT vs 5.6 × 10^9^/L for control, p < 0.01) and at 10 h (6.3 × 10^9^/L for OG + LTT vs 5.9 × 10^9^/L for control, p < 0.05) after the OG + LTT challenge. No significant differences from the control challenge were found for the leukocyte numbers after the OLTT challenge.

An initial increase of % neutrophils was observed with statistically significant differences from baseline within the first 2 hours after all three dietary challenges and the water control challenge (Figure 2). The percentage of neutrophils then gradually declined over time, with the exception of a second peak appearing at 6 h after the OGTT challenge, which was statistically different from baseline, and from the control challenge (62% for OGTT vs 54% for control, p < 0.01). Neutrophil levels in the other groups were not different from the levels after the water control challenge.

In contrast, the percentage of lymphocytes significantly decreased during the first 2 hours after the dietary and water control challenges compared to baseline (Figure 2). The percentage then gradually increased in all groups. Only after the OGTT challenge a second decrease was observed at 6 h, which was statistically different from baseline, and from the control challenge (31% for OGTT vs 38% for control, p < 0.01) thus mirroring the effect observed in the neutrophil population.

**Figure 3:**
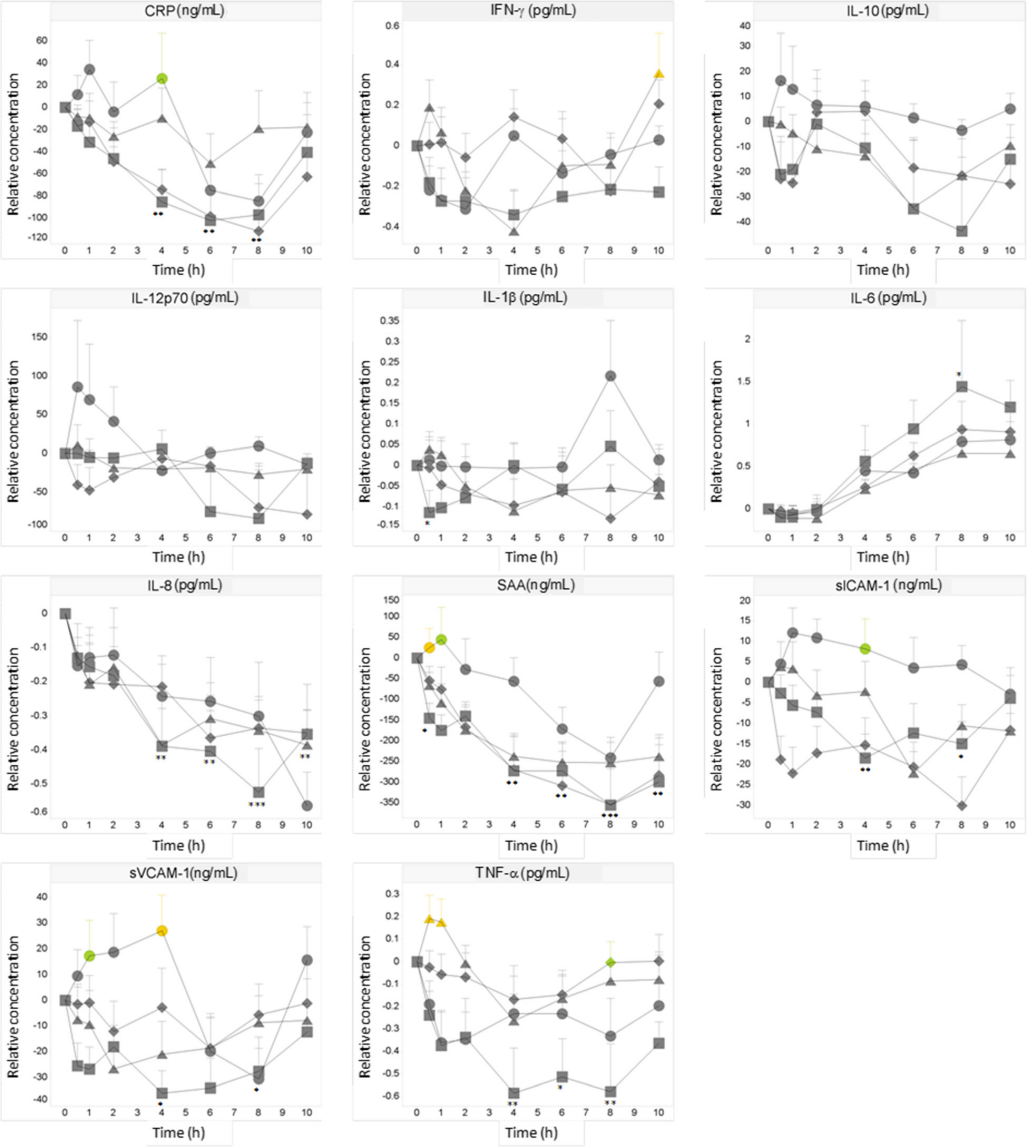
**Effect on inflammatory markers.** Mean ± SEM postprandial relative CRP, IFNγ, IL-10, IL-12p70, IL-1β, IL-6, IL-8, SAA, sICAM-1, sVCAM-1, TNF-α in healthy subjects (N = 14) after ingestion of water (□), OGTT (◊), OLTT (△), OG + LTT (○). ANOVA: OGTT, OLTT, OL + GTT versus water: green p < 0.05, yellow p < 0.01, orange p < 0.001; water versus baseline; * p < 0.05, ** p < 0.01, *** p < 0.001.

Compared to baseline, small statistically significant changes were observed at a few time points in the water control group (i.e. decreases in CRP, IL-8, SAA, TNF-α, sICAM-1, sVCAM-1, E-selectin, P-selectin, thrombomodulin, and increase in IL-6) after OGTT (i.e. decreases in CRP, IL-8, SAA, sICAM-1, increase in IL-6), after OLTT (i.e. decreases in IL-8, SAA, sICAM-1, increase in IL-6) and after OG + LTT (i.e. decreases in IL-8, SAA, thrombomodulin, increase in IL-6) (Figure 3). However, when compared to the control challenge only a limited number of statistically significant differences were observed (Figure 3): after the OG + LTT challenge, increases in sICAM-1 at 4 h (259 ng/mL for OG + LTT vs 239 ng/mL for control, p < 0.01,) sVCAM-1 at 1 h and 4 h (437 and 447 ng/mL for OG + LTT vs 421 and 412 ng/mL for control, p < 0.05 and p < 0.01 respectively), CRP at 4 h (994 ng/mL for OG + LTT vs 908 ng/mL for control, p < 0.05), and SAA at 0.5 h and 1 h (1949 and 1969 ng/mL for OG + LTT vs 1815 and 1785 ng/mL for control, p < 0.01 and p < 0.05, respectively). Furthermore, increases in TNF-α were observed after the OLTT challenge at 0.5 h and 1 h and after the OGTT challenge at 8 h. The statistical significance of these changes (except for CRP) could also be confirmed by the analysis based on repeated measurements. Nevertheless, it should be noted that the increases in the plasma levels of these markers were small. The significant differences were mostly due to decreased levels after the water control and could only be detected when the data were normalized to the baseline levels.

Noteworthy are the observations that were not related to the dietary challenges but to experimental factors such as prolonged fasting and diurnal effects. For example, consistent significant increases in circulating IL-6 levels, and decreases in IL-8 and SAA levels were observed over time after all three dietary challenges, notably also after the water control challenge when compared to baseline (Figure 3). Between-challenge comparisons, however, did not show any significant differences from the control challenge, except for the higher levels of SAA at 0.5 h and 1 h after the OG + LTT challenge.

#### *Oxylipins*

**Figure 4:**
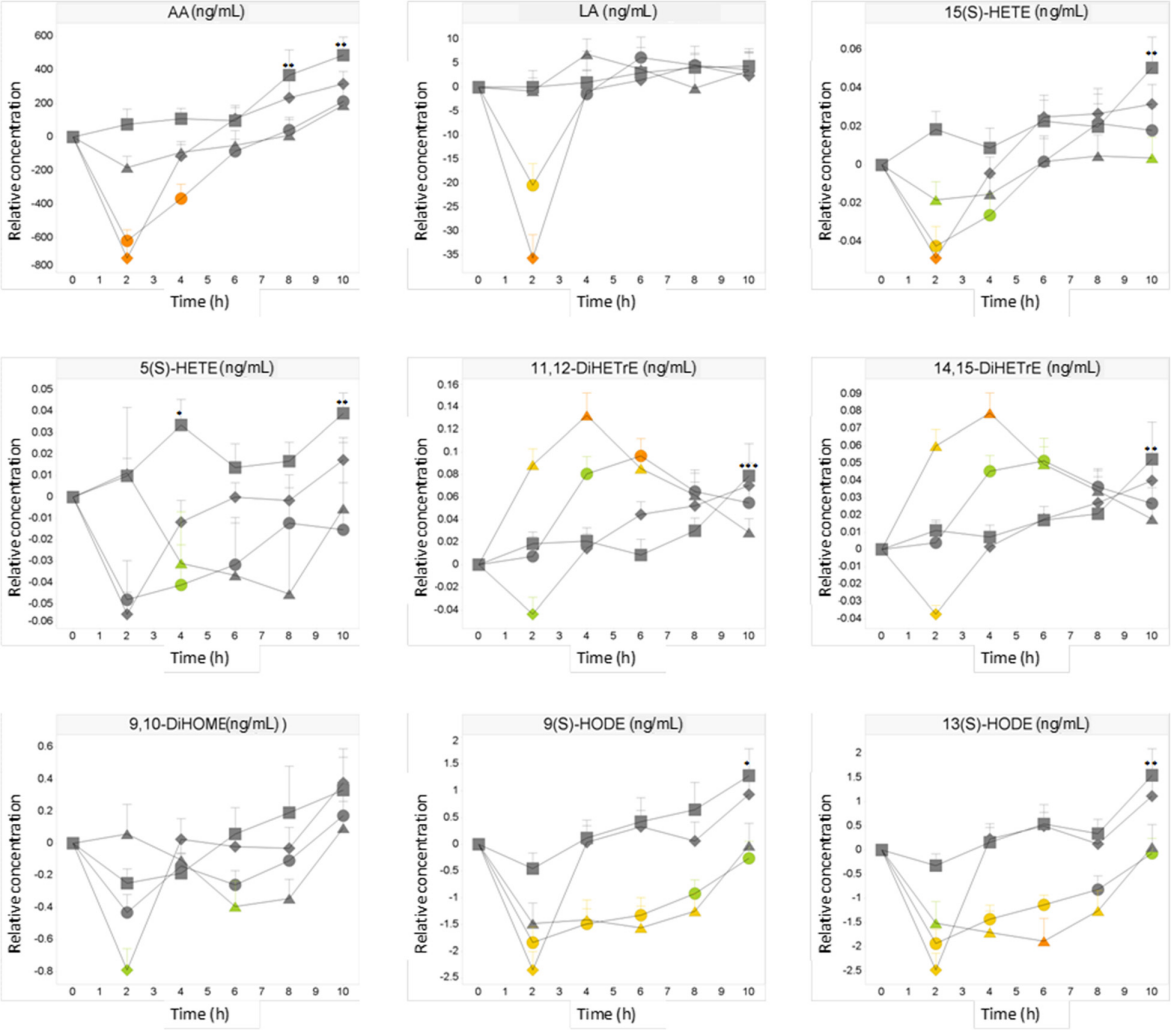
**Effect on oyxlipins.** Mean ± SEM postprandial relative AA, 5(S)-HETE, 15(S)-HETE,11,12-DiHETrE, 14,15-DiHETrE, LA, 9(S)-HODE, 13(S)-HODE, 9,10-DiHOME in healthy subjects (N = 12) after ingestion of water (□), OGTT (◊), OLTT (△), OG + LTT (○). ANOVA: OGTT, OLTT, OL + GTT versus water: green p < 0.05, yellow p < 0.01, orange p < 0.001; water versus baseline; * p < 0.05, ** p < 0.01, *** p < 0.001.

Diverse oxylipins were measured, because these lipid mediators are involved in inflammation and cellular growth processes. In general, the different dietary challenges induced statistically significant effects on the precursor oxylipins AA, LA, DHA and EPA and on a few downstream oxylipin metabolites including the derivatives of LA [9(S)-HODE, 13(S)-HODE, 9,10-DiHOME], AA [5(S)-HETE, 15(S)-HETE,11,12-DiHETrE, 14,15-DiHETrE] and DHA [17(S)-HDoHE, 17-keto-DHA, PGD2]. After the control challenge, significant increases in the precursors AA, DHA and EPA were observed after 8-10 h when compared to baseline (Figure 4, Additional file 1: Figure S2). Some derivatives of these precursors also showed increased concentrations at 10 h, namely the AA-derived oxylipins involved in the LOX pathway [5(S)-HETE, 12(S)-HETE and 15(S)-HETE], in the CYP pathways [11,12-DiHETrE, 14,15-DiHETrE], and the DHA-derived oxylipins [17-(S)-HDoHE and 17keto-DHA]. The concentrations of some AA-derived oxylipins were significantly increased at multiple time points, such as 12(S)-HETE (2, 6 and 10 h) and 5(S)-HETE (6 and 10 h). In comparison to AA and DHA, the precursor LA only tended to be increased, while its derivatives, namely 9(S)-HODE, 13(S)-HODE were significantly increased after 10 h.

After the OGTT challenge, the precursor free fatty acids AA, LA, DHA and EPA were significantly reduced after 2 h when compared to baseline and to control challenge. The concentrations reached control levels after 4 h. The oxylipin derivatives of LA [9(S)-HODE, 13(S)-HODE, 9,10-DiHOME], AA [5(S)-HETE, 15(S)-HETE, 11,12-DiHETrE, 14,15-DiHETrE] and DHA [17-HDoHE, 17-keto DHA] showed similar curves as their precursors LA, AA and DHA, respectively (Figure 4, Additional file 1: Figure S2). The PGD2 response curve differed from its precursor AA: reduced concentrations were observed over the whole time course. The difference from the control values was statistically significant based on repeated measurements.

After the OLTT challenge, the precursors AA, EPA and DHA were reduced over the whole time course with the lowest concentrations at 2 h when compared to the control challenge (Figure 4, Additional file 1: Figure S2). The reductions at 2 h challenge was about 1/3 less than those induced by the OGTT challenge. Interestingly, we observed significant reductions in the derivatives of LA [9(S)-HODE, 13(S)-HODE, 9,10-DiHOME] between 2–8 h while the precursor itself only tended to be decreased. The derivatives of AA [5(S)-HETE and 15(S)-HETE] were reduced over the whole time course similar to its precursor AA when compared to the control challenge. Yet, these reductions were less pronounced when compared to the LA derivatives. In contrast to these metabolites involved in the LOX pathway, the CYP-derived oxylipins [11,12-DiHETrE and 14,15-DiHETRrE] were significantly increased at 2–6 h when compared to control and thus showed an opposite response when compared to their precursor AA.

After the OG + LTT challenge, reductions in the precursors AA, LA, DHA and EPA were observed between 2–8 h (Figure 4, Additional file 1: Figure S2). These concentrations were also the lowest at 2 h, yet slightly higher when compared to the OGTT challenge. The control levels were still not reached at 10 h similar to the OLTT challenge. Only LA showed a fast return (within 2 h) to control levels. The response curves of the oxylipin derivatives may be considered as superpositions of their response curves after the OGTT and OLTT challenges. The concentrations of the LA derivatives [9(S)-HODE, 13(S)-HODE, 9,10-DiHOME] and AA derivatives [5(S)-HETE, 15(S)-HETE] were reduced at most time points with the lowest concentrations at 2 h. The reductions were stronger (based on AUC values) when compared to the OGTT and OLTT challenges alone. Furthermore, the concentrations of the AA-derived 11,12-DiHETrE and 14,15-DiHETrE were increased at 4–6 h. However, these increases were delayed and less strong when compared to the OLTT challenge.

#### *Gene expression*

The effect of different dietary challenges on the RNA transcription in white blood cells was investigated for a selection of genes. The results of the different challenge tests were analyzed against different gene-sets that were specific for this dedicated gene-array as described in Materials and Methods.

Overall, minor gene expression changes were observed upon the different dietary challenges (Additional file 1: Table S5). The water control challenge showed differential gene expression in 13 out of the 85 quantified genes (=15% of genes) (Table 2). Both reduced and increased changes were observed, which were in general below 1.5 fold change, indicating that the gene expression differences were small. The 13 genes were scattered on the different dedicated gene sets such as “PPAR signaling” (4 out of 11), “lipid metabolism related to molecular transport” (3 out of 11), “IL-6 signaling” (3 out of 15) and “IL-10 signaling” (3 out of 17).

**Table 2:** Results of gene expression data

***Genes significantly changed in the control challenge compared to baseline***								
**#**	**Name**	**Symbol**	**GeneID**	**0vs2 h**	**0vs6 h**	**p**		
1	ABCA1	ABCA1	19	1,33	1,40	0.001		
2	FLaP	ALOX5AP	241	1,38	1,12	0.023		
3	CXCR2 = IL-8RB	CXCR2	3579	1,26	1,09	0.042		
4	IL1RA	IL1RN	3557	1,19	1,17	0.009		
5	NLRP3	NLRP3	114548	1,12	1,08	0.011		
6	PPARG	PPARG	5468	-1,33	-1,11	0.004		
7	PTX3	PTX3	5806	-1,37	-1,50	0.013		
8	TLR2 (LPS)	TLR2	7097	-1,13	-1,21	0.030		
9	TNF-α	TNF	7124	1,18	1,18	0.025		
10	CCR6	CCR6	1235	1,02	1,23	0.017		
11	DUSP2	DUSP2	1844	-1,64	-1,55	0.006		
12	FPR2/ALX	FFRA1	2358	1,05	-1,02	0.041		
13	ERK	MAPK1	5594	1,11	1,05	0.022		
***Genes significantly changed in OGTT compared to control challenge***								
**#**	**Name**	**Symbol**	**GeneId**	**p**	**OGTT**		**Control**	
					**0vs2 h**	**0vs6 h**	**0vs2 h**	**0vs6 h**
1	CXCR3	CXCR3	2833	0.009	1,11	-1,07	-1,04	1,23
2	IL10RA	IL10RA	3587	0.016	-1,14	-1,26	-1,12	-1,06
3	INSIG1 (insulin induced gene 1)	INSIG1	3638	0.031	-1,02	-1,06	-1,04	1,01
4	pyruvate dehydrogenase kinase 4	PDK4	5166	0.009	-3,11	1,66	1,11	1,37
5	ITGB1	ITGB1	3688	0.047	-1,09	-1,10	-1,06	1,09
6	lipocalin-2	LCN2	3934	0.0002	-1,07	-1,29	1,10	1,03
7	carnitine-acylcarnitine translocase	SLC25A20	788	0.031	-1.31	1,26	1,08	1,11
***Genes significantly changed in OLTT compared to control challenge***								
**#**	**Name**	**Symbol**	**GeneId**	**p**	**OLTT**		**Control**	
					**0vs2 h**	**0vs6 h**	**0vs2 h**	**0vs6 h**
1	acyl-Coenzyme A dehydrogenase	ACADVL	37	0.029	-1,18	1,37	1,01	1,12
2	CYP4F3	CYP4F3	4051	0.012	1,11	1,20	1,14	1,12
3	ERK	MAPK1	5594	0.035	-1,08	-1,09	1,11	1,05
***Genes significantly changed in OG + LTT compared to control challenge***								
**#**	**Name**	**Symbol**	**GeneId**	**p**	**OG + LTT**		**Control**	
					**0vs2 h**	**0vs6 h**	**0vs2 h**	**0vs6 h**
1	CCR2	CCR2	729230	0.021	1,02	-1,09	-1,11	-1,02
2	Mgl CD301	CLEC10A	10462	0.002	-1,17	-1,01	-1,09	1,18
3	ITGAL CD11a	ITGAL	3683	0.041	-1,10	-1,10	1,02	1,05
5	IL-18	IL18	3606	0.029	-1,01	1,12	-1,18	-1,14
6	CD162 - P-selectin ligand	SELPLG	6404	0.027	1,05	-1,09	1,07	-1,05
7	carnitine-acylcarnitine translocase	SLC25A20	788	0.041	1,08	1,11	-1,25	1,22

Significance is based on repeated measurements.

Seven, three and seven genes responded differently to the OGTT, OLTT and OG + LTT challenges, respectively, when compared to the control challenge (Table 2). These genes did not cluster to specific pathways or biological functions. The strongest effect was found for the gene PDK4 in response to OGTT, which showed a 3-fold decrease at 2 h. Moreover, a significant reduction in IL10RA, a gene that is involved in IL-10 signaling, was found at 6 h after the OGTT challenge. Note that the effect in MAPK after OLTT could be due to differences in baseline values.

Interestingly, despite the significant changes observed for the oxylipin metabolites, only one gene related to oxylipin metabolism, namely CYP2J2, was marginally changed after the OLTT challenge. Other genes encoding enzymes involved in oxylipin metabolism, such as ALOX5, ALOX12, ALOX15 and CYP4F3 were not affected after the dietary challenges.

## Discussion

The goal of this study was to select a challenge model that would allow to study and quantify inflammatory resilience, i.e. the inflammatory stress response after a dietary challenge. Therefore, we assessed the kinetic response of three different dietary challenges and a water control challenge on various markers related to inflammation and metabolic control in 14 healthy males and females.

### Effect on inflammation

None of the dietary challenges induced a well-defined acute inflammatory response when compared to the water control challenge as evidenced by multiple markers directly related to inflammation such as cytokines, vascular markers, CRP, specific oxylipins and genes. Only modest, yet statistically significant increases in leukocytes, sVCAM-1, sICAM-1, SAA and CRP were observed at certain time points after the OG + LTT challenge. Furthermore, subtle and scattered increases in leukocyte numbers and TNF-α were found after the OGTT challenge.

The increase in leucocytes are suggestive for a modest inflammatory response and in agreement with a number of studies showing comparable small increases in leukocyte numbers compared to baseline after dietary challenge [12,14,26-29]. However, only studies by van Oostrom *et al.* included a water control and showed that, next to a generic time-dependent increase in lymphocytes, the increases in leukocytes after lipid, glucose and combined challenges were challenge specific and mostly due to an increase in neutrophils [12,14,29]. Together with our observations, these results are the only firm evidence suggesting that the inflammatory response can be attributed to the dietary challenges and not to other experimental conditions. Yet it is notable, that our observations are less convincing than those by van Oostrom *et al.*, because we only found a significant increase in WBC compared to the water control challenge at 1–2 time points after the OGTT and OG + LTT challenges but not after OLTT alone. It remains unclear why the studies from van Oostrom *et al.* showed clearer effects.

In this study, none of the cytokines and acute-phase proteins were consistently affected by the dietary challenges. Other similar studies in healthy subjects have shown ambiguous results on CRP, TNF-αlpha and IL-6. For example, increases in TNF-α have been reported after high fat loads [15,23], whereas others studies did not find this effect [17,27]. Moreover, increases in postprandial CRP have been reported after OGTT [30] and OLTT [15] challenges, whereas other studies did not observe significant differences in CRP after a high fat challenge [17,27,31]. These different observations are not readily explained by e.g. differences in study population, caloric load or composition of challenges. However, it is noticeable that Derosa *et al.* have demonstrated significant increases in these markers in a much larger population [15,30].

Metabolites mostly indicative of inflammatory processes such as the AA-derived oxylipins involved in the COX pathway [prostaglandins (e.g. PGF2α, PGD2, PGE2) and thromboxanes (e.g. TXB2)] did not show significant effects after the dietary challenges, except for the marginal reduction in PGD2 after the OGTT challenge. Moreover, none of the gene-sets mostly indicative of pro-inflammatory response ['lipid metabolism related to inflammatory response’, 'inflammatory response related to infectious disease’, and 'IL-6 signaling’] or belonging to anti-inflammatory response ['Il-10 signaling’] were significantly regulated by one of the dietary challenges. This is in line with another study, showing no effects on the gene expressions of TNF-α, IL-8 and Nfκb1 upon a high-fat challenge high in saturated fat [32].

### Effect on vascular inflammation

From the three dietary challenges tested in this study, only the OG + LTT challenge induced subtle increases in various vascular inflammatory markers, such as sVCAM-1 and sICAM-1. Effects on sVCAM-1 and sICAM-1 after dietary challenges in healthy subjects are inconsistent, as some studies have reported a significant increase in these markers [8,15,30] in contrast to others that have found no increases [22,33]. Since none of these studies have incorporated a non- or placebo-challenged control group, these observations need to be confirmed in well-controlled studies. The increase in plasma sICAM-1 and sVCAM-1 levels after the OG + LTT challenge compared to the water control as observed in our study has limited value, because the increase was small and partly due to a decrease in the water control. In addition, no between-challenge effects were observed on the other vascular markers, namely sICAM-3, E-selectin, P-selectin, thrombomodulin, leading us to the conclusion that the impact of the dietary challenges on vascular markers of inflammation was limited in this study.

Our results on the oxylipins that are involved in the LOX and CYP pathways may further support the role of endothelial inflammation and vascular functions considering that these metabolites can be incorporated into membranes within vascular tissues in contrast to COX-derived metabolites [34]. It has been suggested that the CYP-derived DiHETrE’s, which were down-regulated after the OGTT challenge and up-regulated after OLTT and OG + LTT challenges in the current study, may be released from the vascular endothelium and lead to vasodilation and vascular smooth-muscle relaxation via stimulation of Ca^2+^-activated K^+^ channels in coronary arteries or via modulation of endothelial NO release [35]. Furthermore, it has been found that the LOX-derived 9-HODE and 13-HODE, which were reduced after all three dietary challenges, can be released by lipoprotein lipase on the endothelium and lead to increased expression of TNF-α, vascular cell adhesion molecule (VCAM) and E-selectin and thus to impaired endothelium-dependent vasodilation [34,36]. 13(S)-HODE has also been reported to inhibit platelet binding to endothelial cells [37] and to have anti-proliferative activity [38].

### Effect on metabolic control

All three dietary challenges clearly induced changes related to metabolic control. In the post-absorptive state, the intake of dietary glucose usually leads to increased serum insulin concentrations followed by reduced lipolysis which is reflected by reductions in free fatty acids (FFA’s) and glycerol concentrations. This explains the consistent reductions of the precursor free fatty acids AA, LA, EPA and DHA 2 h after the OGTT and OL + GTT challenges. As soon as glucose and insulin reach baseline levels, fatty acids from peripheral tissues are released into circulation, as shown by the increase in the precursor oxylipins.

The response curves of the downstream oxylipins after the OGTT challenge were similar to the responses of their precursor free fatty acids suggesting that the regulation of these metabolites relate to insulin signaling. In contrast, after the OLTT challenge, only the CYP epoxygenase products, 11,12-DiHETrE and 14,15-DiHETrE were up-regulated, while the others [e.g. 9(S)-HODE, 13(S)-HODE, 9,10-DiHOME] were down-regulated indicating specific regulation of eicosanoid pathways by this challenge model. The changes of the AA-derived metabolites [DiHETrE’s] may be related to the activity of PPAR’s considering that these transcriptional regulators of lipid and carbohydrate metabolism can be activated by saturated and unsaturated long-chain fatty acids and their eicosanoid (AA) derivatives [39]. The PPAR activation may further modulate the inflammatory response in different immunological and vascular wall cell types [40]. In contrast to the increases in 11,12-DiHETrE and 14,15-DiHETrE after the OLTT challenge, another study including a 4-week treatment with ω-3 fatty acids has resulted in reductions in the AA-derived metabolites DiHETrE’s and increases in EPA- and DHA-derived metabolites involved in the CYP pathway [41]. These differences may account for the anti-inflammatory and pro-resolution effects of ω-3 fatty acids (EPA, DHA) as opposed to the high load of saturated fatty acids with pro-inflammatory properties [6]. They may also reflect a fine balance between EPA, DHA and AA competing for the conversion by CYP enzymes [42].

### Effects that cannot be attributed to the dietary challenges

The inclusion of the water control challenge was crucial to account for factors that were not related to the dietary challenge, such as diurnal variations, prolonged fasting and sample procedure. For example, the gradually decreased concentrations of glucose, insulin and triglycerides and the increased concentrations of several oxylipins observed during the control challenge can be attributed to prolonged fasting. Furthermore, in contrast to widely reported increases in postprandial IL-6 [7,15,17,18,22,27,29,43],[44], we observed a similar increase in IL-6 concentrations in all dietary and the water control challenges suggesting that the effect was not related to the dietary challenges, but rather to local tissue production associated with cannula placement. It has been described that venous cannulation for more than 3 hours may lead to local tissue production of IL-6 [45-47] and thus hamper accurate detection of systemic IL-6. Interestingly, all studies that have reported effects on IL-6 after dietary challenges used a continuous intravenous line [7,15,17,18,22,27,29,43],[44]. Studies that have seen no effects or even decreases in postprandial IL-6 levels either used venapuncture [31] or have not reported the method used for blood sampling [33,48,49]. This suggests that the increase in plasma IL-6 levels may be related to the blood sampling method. Alternatively, the increase in IL-6 may be explained by diurnal variation [50,51], a conclusion also drawn by van Oostrom *et al. *[29] and Tulk *et al. *[18].

## Conclusions

Overall, our results together with evidence from literature indicate that the subtle increase in circulating leukocytes seems the most consistent effect of an inflammatory response after the OGTT and OG + LTT challenge. The changes in cytokine levels that have been observed in some other studies are overall far less consistent and not supported by our data. Moreover, the subtle increases in vascular markers such as sICAM-1 and sVCAM-1 after the OG + LTT challenge are of limited value, because they only became apparent in comparison to the water control challenge which has proved to be essential to control for factors such as diurnal variation, prolonged fasting, and sampling procedure.

It is worth mentioning, that these marginal effects were observed in healthy subjects and after the consumption of a single dose of high fat and/or glucose. It remains to be investigated, whether the threshold for inflammation can be shifted in compromised/diseased population or by sustained dietary intake. In addition, the number of subjects enrolled in this study was low and thus increasing the sample size may translate into higher statistical significance. As alternative to dietary challenges, low-dose lipopolysaccharide (LPS) challenges have been described in literature to elicit time-resolved measurable acute inflammatory responses and thus may be a more appropriate challenge model of low-grade inflammation [52].

Interestingly, all dietary challenges clearly induced changes in several oxylipins that were related to metabolic control and possibly to vascular functions. However, the meaning of these changes often is not clear because of insufficient knowledge on the functions of specific oxylipins. Further studies are required to elucidate the role of oxylipins in metabolic and inflammatory processes.

## Competing interests

The authors state no conflict of interests.

## Authors’ contributions

All authors conceived and designed the study. EB executed the study. MG and UG performed the statistical analyses. SW, DW, MvE, BK, DMJ and FAvD analyzed and interpreted the data. DMJ, SW and DW wrote the paper. JvD, NC, LF, PvdL, HFJH, RA and BvO provided significant advice. All authors read and approved the final manuscript.

## Pre-publication history

The pre-publication history for this paper can be accessed here:

http://www.biomedcentral.com/1755-8794/6/44/prepub

## Supplementary Material

Additional file 1: Table S1 Demographic characteristics of subjects at inclusion. **Table S2.** Parent and product *m/z* values of LC-MS/MS analysis of eicosanoids. The eicosanoids in bold were selected for further processing. **Table S3.** Overview of selected genes related to inflammation and their primers; shaded genes are reference genes; genes in italic and with grey font did not pass quality control or were not considered for data analysis. **Table S4.** Gene-sets related to specialized biological functions and pathways selected from gene-array of a total of 85 genes. **Table S5.** Fold changes of genes. **Figure S1.** Statistical significance of repeated measurements, AUC_min_, AUC_plus_and AUC_total_ of inflammatory markers and clinical paramters (upper panel), oxylipins (middle panel) and gene expression data (lower panel) after OGTT, OLTT and OG + LTT versus control, green p < 0.05, yellow p < 0.01, orange p < 0.001. **Figure S2.** Mean ± SEM postprandial relative EPA, DHA, 17(S)-HDoHE, 17-keto-DHA in healthy subjects (N = 12) after ingestion of water (□), OGTT (◊), OLTT (△), OG + LTT (○). ANOVA: OGTT, OLTT, OL + GTT versus water: green p < 0.05, yellow p < 0.01, orange p < 0.001; water versus baseline; * p < 0.05, ** p < 0.01, *** p < 0.001. Click here for file
