# Supplementary material for: Assessment of inflammatory resilience in healthy subjects using dietary lipid and glucose challenges
Source: BMC Med Genomics. 2013 Oct 27;6:44. doi: 10.1186/1755-8794-6-44 (PMC4015956; doi:10.1186/1755-8794-6-44)
Supplement: Additional file 1: Table S1 — Demographic characteristics of subjects at inclusion. Table S2. Parent and product m/z values of LC-MS/MS analysis of eicosanoids. The eicosanoids in bold were selected for further processing. Table S3. Overview of selected genes related to inflammation and their primers; shaded genes are reference genes; genes in italic and with grey font did not pass quality control or were not considered for data analysis. Table S4. Gene-sets related to specialized biological functions and pathways selected from gene-array of a total of 85 genes. Table S5. Fold changes of genes. Figure S1. Statistical significance of repeated measurements, AUCmin, AUCplusand AUCtotal of inflammatory markers and clinical paramters (upper panel), oxylipins (middle panel) and gene expression data (lower panel) after OGTT, OLTT and OG + LTT versus control, green p < 0.05, yellow p < 0.01, orange p < 0.001. Figure S2. Mean ± SEM postprandial relative EPA, DHA, 17(S)-HDoHE, 17-keto-DHA in healthy subjects (N = 12) after ingestion of water (□), OGTT (◊), OLTT (△), OG + LTT (○). ANOVA: OGTT, OLTT, OL + GTT versus water: green p < 0.05, yellow p < 0.01, orange p < 0.001; water versus baseline; * p < 0.05, ** p < 0.01, *** p < 0.001. [file 1755-8794-6-44-S1.docx]

**Additional Material**

Table S1. Demographic characteristics of subjects at inclusion.

| **Treatment** | **no of obs.** | **mean** | **std** | **min.** | **max.** |
| --- | --- | --- | --- | --- | --- |
| Body weight  [kg] | 14 | 70.2 | 7.6 | 57.7 | 81.2 |
| Height  [m] | 14 | 1.77 | 0.08 | 1.64 | 1.89 |
| Waist circumference  [cm] | 14 | 80 | 8 | 69 | 95 |
| BMI  [kg/m**2] | 14 | 22.3 | 1.0 | 20.5 | 23.6 |
| Heart rate  [beats/min] | 14 | 57 | 9 | 44 | 75 |
| Systolic blood pressure  [mmHg] | 14 | 125 | 19 | 100 | 160 |
| Diastolic blood pressure  [mmHg] | 14 | 79 | 9 | 66 | 103 |
| WHR  [-] | 14 | 0.86 | 0.02 | 0.81 | 0.90 |
| Fat mass I  [kg] | 14 | 14.9 | 4.0 | 9.9 | 25.2 |
| Fat free mass  [kg] | 14 | 55.3 | 8.0 | 45.1 | 68.9 |
| Skeletal muscle mass  [kg] | 14 | 30.7 | 4.8 | 24.8 | 38.7 |
| Visceral fat area  [cm2] | 14 | 76.9 | 21.0 | 37.0 | 105.8 |
| Fat mass II  [%] | 14 | 21.3 | 5.6 | 14.1 | 34.1 |

Table S2. Parent and product *m/z* values of LC-MS/MS analysis of eicosanoids. The eicosanoids in bold were selected for further processing.

| *Compounds* | *Parent (m/z)* | *Product (m/z)* |
| --- | --- | --- |
|  | | |
| 12-HHTrE | 279.0 | 179.3 |
| **LA** | **279.3** |  |
| **13-HODE** | **295.1** | **195.0** |
| **9-HODE** | **295.1** | **171.0** |
| **EPA** | **301.1** | **257.2** |
| **ARA** | **303.1** | **259.2** |
| 12,13-DiHOME | 313.2 | 183.0 |
| **9,10-DiHOME** | **313.2** | **201.0** |
| 15-deoxy-d-12,14-PGJ_2_ | 315.0 | 271.1 |
| 12-HEPE | 317.2 | 179.0 |
| 5-HEPE | 317.2 | 115.0 |
| **11,12-EET** | **319.1** | **167.0** |
| **12-HETE** | **319.1** | **179.2** |
| **5-HETE** | **319.1** | **203.1** |
| **14,15-EET** | **319.1** | **219.2** |
| 5,6-EET | 319.1 | 191.3 |
| 11-HETE | 319.1 | 167.0 |
| 8,9-EET | 319.1 | 167.0 |
| **15-HETE** | **319.2** | **219.1** |
| **20-HETE** | **319.2** | **275.3** |
| 2,3-dinor-8-iso-PGF_2α_ | 325.1 | 237.2 |
| **DHA** | **327.1** | **283.1** |
| 9,10,13-TriHOME * | 329.2 | 171.1 |
| 9,12,13-TriHOME * | 329.2 | 211.1 |
| PGB_2_ | 333.2 | 174.9 |
| LTB_4_ | 335.1 | 194.8 |
| 14,15-DiHETrE | 337.1 | 207.0 |
| **11,12-DiHETrE** | **337.1** | **166.9** |
| 5,6-DiHETrE | 337.1 | 144.8 |
| 8,9-DiHETrE | 337.1 | 127.0 |
| **17-keto-4(z),7(z),10(z),13(z),15(e),19(z) DHA** | **341.5** | **111.0** |
| **17-HDoHE** | **343.1** | **281.4** |
| PGE_3_ | 349.0 | 269.1 |
| PGD_3_ | 349.1 | 269.1 |
| **PGD_2_** | **351.1** | **271.1** |
| **Lipoxin A_4_** | **351.1** | **114.9** |
| 13,14-dihydro-15-keto-PGD_2_ | 351.1 | 175.0 |
| 13,14-dihydro-15-keto-PGE_2_ | 351.1 | 175.1 |
| **PGE_2_** | **351.1** | **271.2** |
| 13,14-dihydro-15-keto-PGF_2α_ | 353.1 | 113.1 |
| 8-iso-PGF_2α_ | 353.1 | 193.0 |
| 11β-PGF_2α_ | 353.1 | 193.0 |
| **PGF_2α_** | **353.1** | **193.0** |
| PGF_2β_ | 353.2 | 193.1 |
| 10,17-DiHDoHE | 359.1 | 152.9 |
| Maresin | 359.6 | 177.1 |
| 19,20-DiHDoPE | 361.1 | 272.7 |
| **TBXB_3_** | **367.1** | **168.9** |
| **TBXB_2_** | **369.1** | **169.0** |
| Resolvin D_2_ | 375.1 | 175.0 |
| Resolvin D_1_ | 375.1 | 140.8 |
| LTE_4_ | 438.0 | 351.0 |
| n-acetyl LTE_4_ | 480.0 | 351.1 |
| LTD_4_ | 495.1 | 142.9 |
| 13-HODE-d4 | 299.2 | 198.2 |
| ARA-d8 | 311.2 | 267.2 |
| 20-HETE-d6 | 325.1 | 281.3 |
| 15-HETE-d8 | 327.2 | 226.1 |
| 14,15-EET-d11 | 330.2 | 268.3 |
| PGB_2_-d4 | 337.1 | 178.9 |
| CUDA | 339.1 | 214.1 |
| LTB_4_-d4 | 339.1 | 197.1 |
| 8,9-DiHETrE-d11 | 348.2 | 127.0 |
| PGE_2_-d4 | 355.1 | 275.1 |
| 13-14-dihydro-15-keto-PGF2_α_-d4 | 357.1 | 187.0 |
| 8-iso-PGF_2α_-d4 | 357.1 | 196.9 |
| 11β-PGF_2α_-d4 | 357.1 | 313.4 |
| PGF_2α_-d4 | 357.1 | 313.4 |
| PGD_2_-d9 | 360.3 | 280.1 |
| TBXB_2_-d4 | 373.1 | 173.0 |
| LTD_4_-d5 | 500.0 | 142.9 |

**Table S3.** Overview of selected genes related to inflammation and their primers; shaded genes are reference genes; genes in italic and with grey font did not pass quality control or were not considered for data analysis.

**Table S3.** Continued.

**Table S4:** Gene-sets related to specialised biological functions and pathways selected from gene-array of a total of 85 genes.

**Table S4:** Continued

**Table S4:** Continued

**Table S4:** Continued

**Table S5:** Fold changes of genes

|  |  | fold change | | | |
| --- | --- | --- | --- | --- | --- |
| Gene |  | control | OGTT | OLTT | OG+LTT |
| ABCA1 | 0vs2h | 1.33 | 1.21 | 1.12 | 1.16 |
| ABCA1 | 0vs6h | 1.40 | 1.33 | 2.47 | 1.34 |
| ACAA2 | 0vs2h | 1.01 | -1.22 | -1.04 | -1.28 |
| ACAA2 | 0vs6h | 1.12 | 1.00 | 1.93 | 1.05 |
| ACADVL | 0vs2h | -1.06 | -1.01 | -1.18 | -1.12 |
| ACADVL | 0vs6h | 1.10 | -1.24 | 1.37 | -1.13 |
| ADIPOR1 | 0vs2h | 1.26 | 1.01 | -1.32 | -1.05 |
| ADIPOR1 | 0vs6h | 1.22 | -1.20 | -1.44 | -1.24 |
| ADIPOR2 | 0vs2h | -1.06 | -1.06 | -1.14 | -1.11 |
| ADIPOR2 | 0vs6h | 1.03 | -1.12 | 1.23 | -1.09 |
| ALOX12 | 0vs2h | 1.05 | -1.04 | -1.15 | -1.10 |
| ALOX12 | 0vs6h | 1.15 | -1.17 | 4.19 | -1.07 |
| ALOX15 | 0vs2h | -1.36 | -1.35 | -1.37 | -1.39 |
| ALOX15 | 0vs6h | -1.25 | -1.81 | 2.33 | -1.09 |
| ALOX5 | 0vs2h | 1.20 | 1.06 | 1.04 | 1.11 |
| ALOX5 | 0vs6h | 1.11 | 1.10 | -1.01 | 1.06 |
| ALOX5AP | 0vs2h | 1.38 | 1.09 | 1.30 | 1.18 |
| ALOX5AP | 0vs6h | 1.12 | 1.29 | 1.07 | 1.14 |
| ATF3 | 0vs2h | -1.19 | -1.18 | 1.03 | -1.22 |
| ATF3 | 0vs6h | -1.07 | -1.27 | 4.08 | -1.08 |
| BCL2A1 | 0vs2h | -1.07 | 1.22 | 1.50 | 1.22 |
| BCL2A1 | 0vs6h | 1.10 | 1.28 | 1.63 | 1.31 |
| CCL2 | 0vs2h | 1.00 | -1.00 | 1.18 | -1.15 |
| CCL2 | 0vs6h | 1.47 | -1.24 | 7.84 | -1.16 |
| CCL5 | 0vs2h | -1.08 | -1.08 | -1.36 | -1.35 |
| CCL5 | 0vs6h | 1.06 | -1.21 | 2.24 | -1.12 |
| CCR1 | 0vs2h | 1.02 | 1.05 | 1.01 | 1.06 |
| CCR1 | 0vs6h | 1.07 | 1.05 | 1.23 | -1.00 |
| CCR2 | 0vs2h | -1.11 | 1.10 | -1.09 | 1.02 |
| CCR2 | 0vs6h | -1.02 | -1.17 | 1.17 | -1.09 |
| CCR3 | 0vs2h | 1.00 | -1.08 | -1.03 | -1.14 |
| CCR3 | 0vs6h | 1.02 | -1.09 | 1.49 | -1.01 |
| CCR5 | 0vs2h | -1.14 | 1.01 | -1.12 | -1.13 |
| CCR5 | 0vs6h | 1.07 | -1.18 | 1.58 | -1.05 |
| CCR6 | 0vs2h | 1.02 | 1.01 | -1.10 | -1.04 |
| CCR6 | 0vs6h | 1.23 | -1.03 | 1.51 | 1.03 |
| CCR7 | 0vs2h | -1.21 | -1.15 | -1.22 | -1.12 |
| CCR7 | 0vs6h | 1.07 | -1.17 | 1.05 | -1.04 |
| CD14 | 0vs2h | 1.05 | 1.08 | -1.09 | 1.08 |
| CD14 | 0vs6h | 1.02 | -1.07 | -1.15 | -1.05 |
| CDC42 | 0vs2h | 1.04 | -1.04 | 1.04 | -1.01 |
| CDC42 | 0vs6h | 1.02 | -1.08 | -1.01 | -1.06 |
| Table S5: continued | | |  |  |  |
| Gene |  | control | OGTT | OLTT | OG+LTT |
| CHUK | 0vs2h | -1.13 | -1.01 | -1.01 | -1.05 |
| CHUK | 0vs6h | 1.03 | 1.01 | 1.41 | 1.03 |
| CLEC10A | 0vs2h | -1.09 | -1.03 | -1.11 | -1.17 |
| CLEC10A | 0vs6h | 1.18 | -1.04 | 2.28 | -1.01 |
| CX3CR1 | 0vs2h | 1.18 | 1.06 | 1.01 | -1.05 |
| CX3CR1 | 0vs6h | 1.18 | -1.06 | 1.19 | 1.03 |
| CXCL10 | 0vs2h | -1.53 | -1.03 | 1.04 | -1.33 |
| CXCL10 | 0vs6h | -1.07 | -1.25 | 3.62 | -1.17 |
| CXCR1 | 0vs2h | 1.15 | 1.08 | 1.05 | 1.10 |
| CXCR1 | 0vs6h | 1.04 | 1.08 | -1.04 | -1.01 |
| CXCR2 | 0vs2h | 1.26 | 1.07 | 1.11 | 1.12 |
| CXCR2 | 0vs6h | 1.09 | 1.07 | -1.01 | 1.01 |
| CXCR3 | 0vs2h | -1.04 | 1.11 | -1.22 | -1.14 |
| CXCR3 | 0vs6h | 1.23 | -1.07 | 3.63 | -1.06 |
| CYP2J2 | 0vs2h | -1.16 | 1.13 | -1.01 | -1.13 |
| CYP2J2 | 0vs6h | 1.05 | -1.13 | 3.91 | -1.16 |
| CYP4F3 | 0vs2h | 1.14 | 1.12 | 1.11 | 1.13 |
| CYP4F3 | 0vs6h | 1.12 | 1.27 | 1.20 | 1.08 |
| DUSP2 | 0vs2h | -1.64 | -1.50 | -2.05 | -1.76 |
| DUSP2 | 0vs6h | -1.55 | -1.90 | 1.51 | -1.56 |
| EPHX2 | 0vs2h | -1.15 | -1.12 | -1.21 | -1.20 |
| EPHX2 | 0vs6h | 1.06 | -1.09 | 1.27 | -1.10 |
| FFAR2 | 0vs2h | 1.11 | 1.28 | 1.12 | 1.24 |
| FFAR2 | 0vs6h | 1.24 | 1.23 | 1.34 | 1.18 |
| FPR2 | 0vs2h | 1.05 | 1.05 | 1.03 | 1.06 |
| FPR2 | 0vs6h | -1.02 | 1.02 | 1.01 | -1.03 |
| GAPDH | 0vs2h | -1.04 | 1.00 | -1.13 | 1.03 |
| GAPDH | 0vs6h | -1.09 | -1.10 | -1.13 | -1.05 |
| HIF1A | 0vs2h | 1.02 | -1.13 | -1.01 | 1.01 |
| HIF1A | 0vs6h | 1.10 | 1.14 | 1.14 | 1.06 |
| HMOX1 | 0vs2h | -1.15 | -1.22 | -1.21 | -1.22 |
| HMOX1 | 0vs6h | -1.07 | -1.25 | 1.08 | -1.10 |
| IKBKB | 0vs2h | -1.11 | -1.05 | -1.13 | -1.05 |
| IKBKB | 0vs6h | 1.07 | -1.09 | 1.05 | -1.05 |
| IL10RA | 0vs2h | -1.12 | -1.14 | -1.26 | -1.13 |
| IL10RA | 0vs6h | -1.06 | -1.26 | -1.20 | -1.14 |
| IL10RB | 0vs2h | 1.05 | 1.01 | 1.03 | 1.06 |
| IL10RB | 0vs6h | 1.07 | 1.15 | 1.06 | 1.09 |
| IL18 | 0vs2h | -1.18 | 1.02 | 1.10 | -1.01 |
| IL18 | 0vs6h | -1.14 | 1.04 | 1.07 | 1.12 |
| IL1B | 0vs2h | -1.06 | -1.00 | 1.07 | -1.01 |
| IL1B | 0vs6h | 1.01 | -1.04 | -1.03 | -1.01 |
| IL1RN | 0vs2h | 1.19 | 1.23 | 1.27 | 1.11 |
| IL1RN | 0vs6h | 1.17 | 1.30 | 1.12 | 1.05 |
| Table 5S: continued | | |  |  |  |
| Gene |  | control | OGTT | OLTT | OG+LTT |
| IL6R | 0vs2h | 1.13 | 1.05 | -1.02 | 1.07 |
| IL6R | 0vs6h | 1.10 | 1.13 | -1.06 | 1.01 |
| INSIG1 | 0vs2h | -1.04 | -1.02 | -1.08 | -1.40 |
| INSIG1 | 0vs6h | 1.01 | -1.06 | 1.02 | -1.21 |
| ITGA4 | 0vs2h | -1.11 | -1.07 | -1.14 | -1.15 |
| ITGA4 | 0vs6h | -1.04 | -1.17 | -1.00 | -1.08 |
| ITGAL | 0vs2h | 1.02 | -1.04 | -1.24 | -1.10 |
| ITGAL | 0vs6h | 1.05 | -1.14 | -1.13 | -1.10 |
| ITGB1 | 0vs2h | -1.06 | -1.09 | -1.08 | -1.10 |
| ITGB1 | 0vs6h | 1.09 | -1.10 | 1.08 | -1.01 |
| ITGB2 | 0vs2h | 1.07 | 1.00 | -1.18 | 1.09 |
| ITGB2 | 0vs6h | 1.07 | -1.07 | -1.22 | -1.03 |
| LCN2 | 0vs2h | 1.10 | -1.07 | -1.23 | -1.13 |
| LCN2 | 0vs6h | 1.03 | -1.29 | -1.42 | -1.39 |
| MAPK1 | 0vs2h | 1.11 | 1.06 | -1.08 | 1.04 |
| MAPK1 | 0vs6h | 1.05 | 1.05 | -1.09 | -1.05 |
| MAPK6 | 0vs2h | -1.07 | 1.02 | 1.07 | -3.05 |
| MAPK6 | 0vs6h | 1.06 | -1.01 | 1.18 | -2.74 |
| MAPK8 | 0vs2h | -1.04 | -1.00 | 1.00 | -1.41 |
| MAPK8 | 0vs6h | -1.02 | -1.01 | 1.04 | -1.35 |
| MMP9 | 0vs2h | 1.66 | 1.21 | 1.46 | 1.38 |
| MMP9 | 0vs6h | 1.24 | 2.02 | -1.02 | 1.33 |
| NAB2 | 0vs2h | 1.00 | -1.04 | -1.13 | -1.41 |
| NAB2 | 0vs6h | -1.08 | -1.14 | -1.20 | -1.59 |
| NCF1 | 0vs2h | 1.17 | 1.00 | 1.15 | 1.14 |
| NCF1 | 0vs6h | 1.09 | 1.13 | -1.05 | 1.07 |
| NFE2L2 | 0vs2h | -1.09 | -1.05 | -1.06 | -1.03 |
| NFE2L2 | 0vs6h | -1.08 | -1.11 | -1.05 | -1.06 |
| NFKB1 | 0vs2h | -1.03 | -1.05 | -1.20 | -1.09 |
| NFKB1 | 0vs6h | 1.03 | -1.09 | -1.08 | -1.14 |
| NFKB2 | 0vs2h | -1.01 | -1.06 | -1.15 | -1.07 |
| NFKB2 | 0vs6h | 1.08 | 1.00 | -1.16 | -1.08 |
| NLRP3 | 0vs2h | 1.12 | 1.18 | 1.08 | 1.02 |
| NLRP3 | 0vs6h | 1.08 | 1.05 | -1.08 | -1.17 |
| PDE4B | 0vs2h | -1.01 | -1.07 | -1.03 | 1.03 |
| PDE4B | 0vs6h | 1.00 | -1.01 | 1.00 | -1.03 |
| PDK4 | 0vs2h | 1.11 | -3.11 | -1.07 | -3.04 |
| PDK4 | 0vs6h | 1.37 | 1.66 | 1.63 | -1.02 |
| PLA2G4A | 0vs2h | -1.03 | 1.20 | 1.25 | 1.12 |
| PLA2G4A | 0vs6h | -1.06 | 1.15 | 1.21 | 1.01 |
| PPARA | 0vs2h | -1.18 | -1.11 | -1.32 | -1.25 |
| PPARA | 0vs6h | -1.12 | -1.19 | -1.16 | -1.21 |
| PPARG | 0vs2h | -1.33 | -1.04 | -1.20 | -1.20 |
| PPARG | 0vs6h | -1.11 | -1.11 | 1.09 | 1.05 |
| Table 5S: continued | |  |  |  |  |
| Gene |  | control | OGTT | OLTT | OG+LTT |
| PTGDS | 0vs2h | -1.01 | -1.05 | -1.28 | -1.21 |
| PTGDS | 0vs6h | -1.10 | -1.18 | -1.19 | -1.13 |
| PTGES2 | 0vs2h | -1.08 | 1.04 | -1.23 | -1.03 |
| PTGES2 | 0vs6h | -1.07 | -1.11 | -1.23 | -1.05 |
| PTGS1 | 0vs2h | 1.06 | 1.03 | -1.25 | 1.01 |
| PTGS1 | 0vs6h | 1.02 | -1.10 | -1.37 | -1.07 |
| PTGS2 | 0vs2h | -1.13 | -1.09 | -1.06 | -1.10 |
| PTGS2 | 0vs6h | -1.09 | -1.03 | -1.03 | -1.02 |
| PTX3 | 0vs2h | -1.37 | -1.07 | -1.17 | 1.07 |
| PTX3 | 0vs6h | -1.50 | -1.44 | -1.14 | -1.09 |
| RAGE | 0vs2h | 1.77 | 3.24 | 1.54 | 1.06 |
| RAGE | 0vs6h | 3.29 | 2.30 | 1.09 | 1.25 |
| RELA | 0vs2h | 1.10 | -1.00 | -1.10 | 1.05 |
| RELA | 0vs6h | 1.10 | 1.00 | -1.13 | -1.02 |
| RETN | 0vs2h | -1.10 | 1.16 | -1.00 | 1.03 |
| RETN | 0vs6h | -1.17 | -1.03 | -1.10 | 1.05 |
| SELL | 0vs2h | 1.11 | 1.03 | 1.10 | 1.16 |
| SELL | 0vs6h | 1.16 | 1.22 | 1.14 | 1.22 |
| SELPLG | 0vs2h | 1.07 | -1.03 | -1.07 | 1.05 |
| SELPLG | 0vs6h | -1.05 | -1.09 | -1.22 | -1.09 |
| SIRT1 | 0vs2h | -1.09 | -1.12 | -1.12 | 1.01 |
| SIRT1 | 0vs6h | -1.04 | -1.12 | 1.09 | 1.02 |
| SLC25A20 | 0vs2h | 1.08 | -1.31 | -1.09 | -1.25 |
| SLC25A20 | 0vs6h | 1.11 | 1.26 | 1.15 | 1.22 |
| SOD1 | 0vs2h | -1.10 | -1.05 | -1.01 | -1.06 |
| SOD1 | 0vs6h | 1.05 | -1.09 | 1.07 | 1.11 |
| SOD2 | 0vs2h | 1.19 | 1.07 | 1.07 | 1.17 |
| SOD2 | 0vs6h | 1.13 | 1.07 | -1.03 | 1.10 |
| TBXAS1 | 0vs2h | 1.08 | 1.10 | 1.03 | 1.13 |
| TBXAS1 | 0vs6h | -1.01 | -1.01 | -1.17 | -1.03 |
| TLR2 | 0vs2h | -1.13 | -1.01 | -1.08 | 1.05 |
| TLR2 | 0vs6h | -1.21 | -1.25 | -1.22 | -1.13 |
| TLR4 | 0vs2h | 1.09 | 1.14 | 1.15 | 1.20 |
| TLR4 | 0vs6h | 1.01 | 1.22 | -1.03 | 1.18 |
| TNF | 0vs2h | 1.18 | 1.30 | 1.21 | 1.23 |
| TNF | 0vs6h | 1.18 | 1.23 | 1.13 | 1.34 |
| TNFAIP3 | 0vs2h | -1.20 | -1.21 | -1.42 | -1.13 |
| TNFAIP3 | 0vs6h | -1.18 | -1.27 | -1.21 | -1.07 |
| TNFRsf1A | 0vs2h | 1.07 | 1.04 | -1.01 | 1.12 |
| TNFRsf1A | 0vs6h | 1.01 | 1.02 | -1.15 | -1.01 |
| TNFRsf1B | 0vs2h | 1.05 | 1.05 | -1.11 | 1.07 |
| TNFRsf1B | 0vs6h | 1.01 | 1.04 | -1.16 | 1.04 |
| YWHAZ | 0vs2h | 1.05 | -1.04 | 1.01 | 1.02 |
| YWHAZ | 0vs6h | 1.11 | -1.02 | 1.10 | 1.06 |

### Figures

|  |
| --- |
|  |
|  |

Figure S 1. Statistical significance of repeated measurements, AUC_min_, AUC_plus_and AUC_total_ of inflammatory markers and clinical paramters (upper panel), oxylipins (middle panel) and gene expression data (lower panel) after OGTT, OLTT and OG+LTT versus control, green p<0.05, yellow p<0.01, orange p<0.001.

|  |  |  |
| --- | --- | --- |
|  |  |  |

Figure S 2. Mean ± SEM postprandial relative EPA, DHA, 17(S)-HDoHE, 17-keto-DHA in healthy subjects (N=12) after ingestion of water (□), OGTT (◇), OLTT (△), OG+LTT (○). ANOVA: OGTT, OLTT, OL+GTT versus water: green p<0.05, yellow p<0.01, orange p<0.001; water versus baseline; * p<0.05, ** p<0.01, *** p<0.001.
